# The Major Cat Allergen Fel d 1 Binds Steroid and Fatty Acid Semiochemicals: A Combined In Silico and In Vitro Study

**DOI:** 10.3390/ijms21041365

**Published:** 2020-02-18

**Authors:** Cécile Bienboire-Frosini, Rajesh Durairaj, Paolo Pelosi, Patrick Pageat

**Affiliations:** 1 Department of Molecular Biology and Chemical Communication (D-BMCC), Research Institute in Semiochemistry and Applied Ethology (IRSEA), Quartier Salignan, 84400 Apt, France; r.durairaj@group-irsea.com; 2 Austrian Institute of Technology GmbH, Biosensor Technologies, Konrad-Lorenzstraße, 3430 Tulln, Austria; ppelosi.obp@gmail.com; 3 Department of Chemical Ecology (D-EC), Research Institute in Semiochemistry and Applied Ethology (IRSEA), Quartier Salignan, 84400 Apt, France; p.pageat@group-irsea.com

**Keywords:** secretoglobin, odorant-binding protein, chemical communication, pheromone, N-phenyl-1-naphthylamine, in silico docking, molecular modeling, protein–ligand interactions, 2D interaction maps, ligand-binding assays

## Abstract

The major cat allergen Fel d 1 is a tetrameric glycoprotein of the secretoglobin superfamily. Structural aspects and allergenic properties of this protein have been investigated, but its physiological function remains unclear. Fel d 1 is assumed to bind lipids and steroids like the mouse androgen-binding protein, which is involved in chemical communication, either as a semiochemical carrier or a semiochemical itself. This study focused on the binding activity of a recombinant model of Fel d 1 (rFel d 1) towards semiochemical analogs, i.e., fatty acids and steroids, using both in silico calculations and fluorescence measurements. In silico analyses were first adopted to model the interactions of potential ligands, which were then tested in binding assays using the fluorescent reporter N-phenyl-1-naphthylamine. Good ligands were fatty acids, such as the lauric, oleic, linoleic, and myristic fatty acids, as well as steroids like androstenone, pregnenolone, and progesterone, that were predicted by in silico molecular models to bind into the central and surface cavities of rFel d 1, respectively. The lowest dissociation constants were shown by lauric acid (2.6 µM) and androstenone (2.4 µM). The specific affinity of rFel d 1 to semiochemicals supports a function of the protein in cat’s chemical communication, and highlights a putative role of secretoglobins in protein semiochemistry.

## 1. Introduction

The major cat allergen Fel d 1 is a secreted globular protein belonging to the family of secretoglobins. It is produced in large amounts in various anatomical areas of cats, such as the salivary, lacrimal, and sebaceous glands from the facial area, skin, and anal sacs [1,2,3,4]. The secretion of Fel d 1 is under androgen control [5]. Fel d 1 is a 35–38 kDa tetrameric glycoprotein composed of two heterodimers with a dimerization interface. Each heterodimer consists of two polypeptide chains encoded by independent genes and linked by three disulfide bridges. Chain 1 is made of 70 residues, and chain 2 of 90 or 92 residues [4,6,7]. Chain 2 contains an N-linked oligosaccharide composed of triantennary glycans [8].

Structural and immunological Fel d 1 polymorphisms have long been described in samples from various origins [8,9,10]. Fel d 1 is a resistant protein easily airborne that is abundantly found in different indoor environments [11,12]. Despite its high abundance and the serious health issues associated with this protein, the biological function of Fel d 1 remains unclear [4].

For other members of the secretoglobin family, different biological roles have been suggested, mainly related to immunoregulation [13,14,15,16], but also in chemical signaling [17,18,19]. Also for Fel d 1, a role in intra-species chemical communication has been proposed based on the fact that the protein is produced in the same areas known to release the cat semiochemicals, including the facial area, the podial complex, and the perianal zone, which contain glands that secrete chemical cues involved in cat territorial marking and/or social communication [20,21]. Besides, Fel d 1 immunological features have been linked to cat sex and behavior [22]. From a structural perspective, Fel d 1 also displays interesting features regarding ligand binding capabilities due to the presence of two internal cavities [23]. Structural similarities between Fel d 1 and another secretoglobin involved in mice mate selection and communication, the mouse salivary androgen-binding protein (ABP) [18], have been previously described [24,25]. Binding of some steroids to members of the secretoglobin family was previously reported, involving interactions with their central hydrophobic cavity [19,23,26]. In particular, a recent paper extensively describes the evolutionary divergence, functional sites, and surface structural resemblance between Fel d 1 and ABP, suggesting that the first protein could be involved in semiochemical transport/processing in intra-species communication [25]. However, so far, no experimental evidence has been provided on the capability of Fel d 1 to bind semiochemicals.

Production of recombinant Fel d 1 (rFel d 1) has been challenging in the past since the two chains are encoded by two different genes, and attempts to refold them in a correct (i.e., with retained disulfide formations) and stable way failed [4,27]. Hence, some authors proposed a rFel d 1 construct made of chain 1 linked to chain 2 via a flexible peptide linker of the (GGGGS)n type [28], which minimizes the steric hindrance between the two fusion partners, since the small size of these amino acids provides flexibility, and allows for mobility of the connecting functional domains [29]. The rFel d 1 displayed similar biological and structural properties (notably the disulfide pairing) to its natural counterparts [8,28].

In the current study, we have investigated the binding properties of this recombinant form of Fel d 1 produced in a *Pichia pastoris* clone with the N-glycosylation site N103 mutated and commercially available (INDOOR Biotechnologies) [30]. As a first step to verify the hypothesis of a role of Fel d 1 in chemical communication, we focused on putative ligands that had already been described as semiochemicals in the domestic cat [21,31], i.e., some fatty acids and their derivatives found in the composition of the feline facial pheromone F3 and the maternal cat appeasing pheromone. The feline facial pheromone F3 has been shown to promote calmness and reduce stress with its related undesirable consequences in cats, such as urine spraying and marking behavior [32,33,34,35]. The maternal cat appeasing pheromone has been shown to have appeasing effects and to facilitate social interactions in cats [36,37]. We have also tested some steroids since several secretoglobins have been experimentally shown to bind steroid hormones, including pig pheromaxein, rabbit uteroglobin, mouse salivary ABP, and rat prostatein [19,38,39,40]. To determine the affinity of these putative ligands and structurally characterize their interactions with rFel d 1, we used a double approach combining in silico analysis (molecular docking) with in vitro fluorescence binding assays.

## 2. Results

### 2.1. In Silico Molecular Docking of Fel d 1 with Putative Ligands

As a first approach to evaluate the binding properties of rFel d 1, we performed docking simulations of flexible ligands into the binding pocket of a rigid binding protein, represented as a grid box [41]. The collected data are reported in Table 1.

Among the 15 fatty acids tested with rFel d 1, lauric, myristic, oleic, and linoleic acids were the best ligands based on their H-bond interactions, docking energy values, and binding frequency. In particular, lauric acid showed the highest frequency of binding with a free energy of −5.84 kcal/mol. The same compound also exhibited the lowest total intermolecular energy of −8.58 kcal/mol. Myristic, linoleic, and oleic acids were moderate ligands with free binding energies of −3.35, −2.95, and −2.82 kcal/mol, respectively. Furthermore, we observed non-bonded interactions (van der Waals and electrostatic), and pi-interactions with all the fatty acids tested.

The second series of chemicals tested includes several steroids. Among these, androstenone showed the maximum frequency of binding as well as the best free binding energy (−5.84 kcal/mol) with one H-bond interaction (S138) in rFel d 1. The behavior of androstenedione was very similar, with a binding energy of −5.83 kcal/mol, but this ligand exhibited a lower frequency of binding without H-bond interaction. On the other hand, progesterone and pregnenolone showed approximately 60% of the binding frequency, with binding energies comparable to those of androstenone and androstenedione. Pregnenolone and progesterone exhibited similar H-bond interactions (Thr76) but different from those of androstenone (S138). Furthermore, Tyr81 and Phe85 were often present as alkyl/pi-alkyl interactions in the steroid compounds.

Finally, our docking simulation predicted high binding activity of the fluorescent probe N-phenyl-1-naphthylamine (1-NPN) in the same range as those for fatty acids and steroids. Specifically, this compound has two potential binding localizations, i.e., in the central and in the surface binding cavities of rFel d 1. Conversely, some fatty acids and structurally related compounds (long-chain alcohols, aldehydes, ester, and amides), as well as few steroids, did not qualify as good ligands in docking simulations and fluorescent probe displacement (Table 1).

Overall, in silico screening indicated as the best potential ligands for the protein some fatty acids and steroids, which were further tested in fluorescence competitive binding assays.

### 2.2. Fluorescence Binding Studies

**Figure 1:**
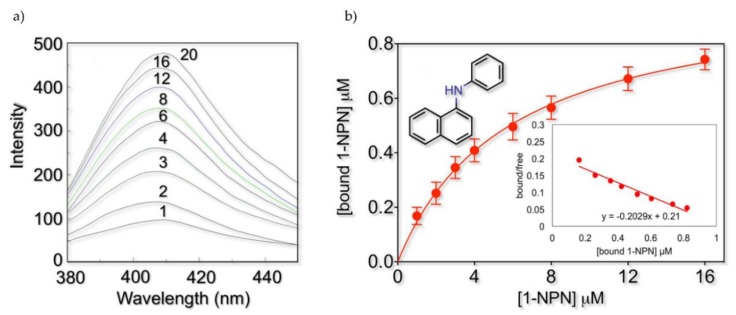
1-NPN binding to rFel d 1. To a 1-µM solution of the protein in 50 mM Tris-HCl, pH 7.4, aliquots of 1 mM solution of 1-NPN in methanol were added to final concentrations of 1–20 µM. (**a**) The representative emission curves experimentally obtained. No significant fluorescence emission was recorded in the same conditions with the protein alone (not shown). (**b**) The saturation binding curve obtained from the average of three experiments. Data were analyzed with GraphPad software and gave a value of 5.8 µM for the binding constant (SD 0.62). The relative Scatchard plot (inset) shows a linear behavior, apparently indicating the presence of a single binding site without cooperativity effects.

The rFel d 1 binds the fluorescent probe 1-NPN, producing a blueshift in the emission spectrum. Similarly to odorant-binding proteins (OBPs) and chemosensory proteins (CSPs) [42], the emission maximum occurs at 407 nm and is accompanied by a strong increase in fluorescence intensity. Figure 1 reports the actual emission spectra obtained with a rFel d 1 concentration of 1 µM and the relative binding curve obtained after processing the data with the GraphPad Software, Inc., giving a dissociation constant of 5.8 µM. Scatchard analysis confirmed the presence of a single binding site on the protein without any cooperativity effect and yielded a dissociation constant K_1-NPN_ value of 4.8 µM. We also tested other fluorescent probes (2-NPN, 1-AMA (1-aminoanthracene), 1,8-ANS (8-anilinonaphtalene sulfonic acid), but none proved to perform better than 1-NPN (data not shown).

**Figure 2:**
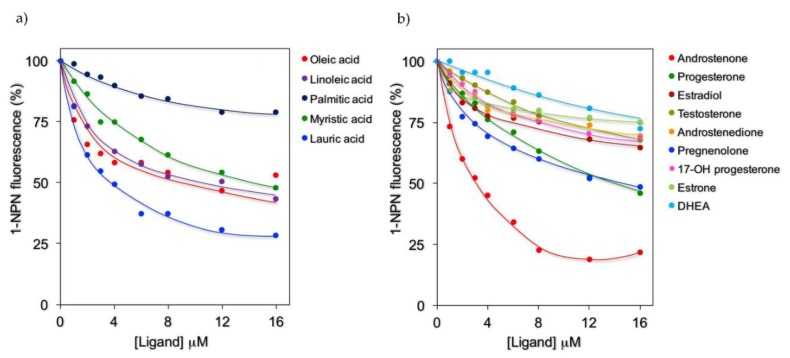
Competitive binding of selected fatty acids (**a**) and steroids (**b**) to rFel d 1. Fluorescence emission spectra were recorded at 25 °C in the presence of 1 µM of rFel d 1 and 2 μM of 1-NPN; excitation and emission wavelengths were 337 and 407 nm, respectively. Fluorescence of probe-protein complexes in the absence of a competitor was normalized to 100%.

Among the 28 putative ligands, 5 fatty acids and 9 steroids were predicted to possibly interact with rFel d 1 based on the initial 1-NPN displacement screening (Table 1). These compounds were therefore tested in competitive binding experiments with 1-NPN and their displacement curves are reported in Figure 2. Table 2 lists the IC50 values for the best ligands, together with their dissociation constants. These were calculated using the value for 1-NPN (K_D_ 5.8 µM; SD 0.62), obtained with GraphPad software, more reliable than that evaluated from the Scatchard plot. Among the fatty acids, lauric acid exhibited the best affinity to rFel d 1 (Kd = 2.6 µM), while oleic, linoleic, and myristic acids displayed only moderate to low affinities, and palmitic acid proved to be the weakest ligand. Among the steroids, the strongest ligand was androstenone (Kd = 2.4 µM), followed by progesterone and pregnenolone. These results are in agreement with the in silico docking predictions.

### 2.3. Visualization of Molecular Interactions

**Figure 3:**
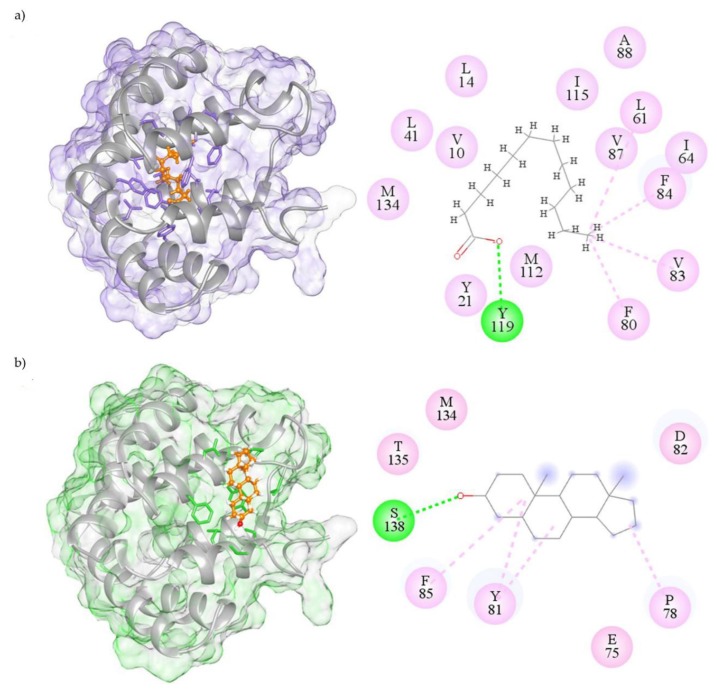
Molecular residue interactions of Fel d 1 with the best ligands, lauric acid (**a**) and androstenone (**b**). The interactions are shown in molecular ligand binding view (surface mesh) with a 2D-interaction map of the selective best-fitting ligands to the central and surface binding cavities of Fel d 1. The 2D map reports H-bond interactions in green color and hydrophobic interactions (van der Waals and alkyl/pi-alkyl) in pink color. All the amino acid residue interactions within 4 Å from the ligand are shown.

To visualize the possible binding modes of the best ligands to rFel d 1, molecular models and 2D molecular interaction maps were built and are shown in Figure 3. Lauric acid is predicted to bind in the central hydrophobic cavity of Fel d 1, where the strongest H-bond interaction occurs between the phenolic hydrogen of Tyr119 and the oxygen of lauric acid (Figure 3a). Androstenone, instead, is predicted to bind on the surface binding cavity of Fel d 1 and shows an H-bond between the Ser138 OH and the carbonyl group of the ligand (Figure 3b).

## 3. Discussion

On the basis of ligand-binding experiments, using the displacement of a fluorescent probe, and in silico docking simulations, we have shown that a recombinant form of Fel d 1 binds with good affinities some fatty acids and steroids, the best ligands being lauric acid and androstenone (Kd = 2.6 and 2.4 µM, respectively). Lauric acid is a component of the mixture of fatty acids described as the cat appeasing pheromone having effects on cats’ social interactions [36,37], together with oleic, linoleic, and myristic acids, which also showed some affinity to rFel d 1. Androstenone is a volatile steroid pheromone found in high concentrations in the saliva of male pigs and triggers attraction/standing responses in estrous females [43]. Interestingly, some authors have also characterized the binding of isoforms from both native and recombinant pig OBP to fatty acids with appeasing effects and some steroids, indicating the biological relevance of these ligands in chemical communication [44,45]. Although the data here presented were obtained with a recombinant form of Fel d 1, they still support a role of this protein in the cat’s chemical communication, probably as a semiochemicals carrier, similar in its function to OBPs [46].

From a structural perspective, molecular docking suggests that, among good ligands, fatty acids, except for linoleic acid, bind in the internal/central cavity of rFel d 1, while steroids bind in the cavity at the surface of the protein. 1-NPN, however, is predicted to fit into both cavities. This last observation could explain how both fatty acids and steroids can displace the fluorescent probe. The same fact might also account for the observation that lauric acid and androstenone, the two best ligands, fail to completely quench 1-NPN fluorescence, showing asymptotic behavior at concentrations much higher than zero. The same phenomenon might occur with other ligands but would not be clearly visible due to their much lower affinities. The presence of two binding sites for 1-NPN might contrast with the linear Scatchard plot. However, if the two sites present similar affinities for 1-NPN and there is no cooperativity effect, the Scatchard analysis would still produce a linear behavior. Incidentally, it is worth noting that, to the best of our knowledge, this is the first report of using a hydrophobic fluorescent probe (1-NPN) to monitor the binding activity of a secretoglobin family member. This probe, therefore, represents a useful tool for monitoring ligand binding properties with other proteins of the family and investigating their putative involvement in chemical signaling [47].

Looking more closely at the residual interactions, the in silico predictions revealed that fatty acids would mainly interact with the hydrophobic residues Val10, Phe13, Leu14, Tyr21, Phe80, Phe84, Val87, Met112, Tyr119, Asp130, and Met134. In the same way, the amino acids Glu75, Thr76, Pro78, Tyr81, Asp82, Phe85, Gly131, Thr135, and Ser138 (all corresponding to only chain 2 residues of the natural Fel d 1 [48]) displayed predicted hydrophobic interactions with the steroids. The present results are in agreement with the few steroid interactions previously described in Fel d 1 [23]. In particular, Tyr21 was previously reported to be highly conserved in several secretoglobins [25] and possibly involved in ligand binding [49,50]. Phe6 was also predicted to interact with ligands [50]. These previous reports suggested that both these amino acids could be important for a function of the protein in chemical communication. Likewise, in the present study, we predicted that Tyr21, Phe84, and Tyr119 could interact with fatty acids, while Tyr81 and Phe85 could interact with steroids.

A limitation of the in silico study is that we used the docking protocol, which is a static or quasi-static method, to obtain the structure of the various Fel d 1-ligand complexes. Using a scoring function that is meant to reproduce the binding affinity in terms of free binding energy, these structures are ranked to reveal the best-fit ligands in a way comparable to the rank based on experimental data [51]. Although the molecular docking free energy differences estimations are fast, simple, and useful for the screening of ligands, they are not the most precise ones (compared to the free binding energies determined by molecular dynamics simulations for instance) due to the absence of mobility or the absence of an explicit solvation of the system [51]. Nevertheless, here, we also considered other computational factors like binding frequencies and residue interactions before concluding about the results of the in silico screening displayed in Table 1. Moreover, these results were further confirmed by in vitro experiments.

A limitation of the in vitro study is that we used a recombinant model of the native Fel d 1, in which a peptide segment was introduced as a linker between the two subunits in the place of disulfide bridges. However, the recombinant and native Fel d 1 secondary structures were found to be similar based on circular dichroism [52]. Most importantly, the disulfide pairing of recombinant Fel d 1 corresponds with that of the native Fel d 1 [8,52]. Therefore, the peptide link in rFel d 1 seems not to introduce major differences in the overall folding of the protein. Whereas the overall structures of native Fel d 1 and of its recombinant are reasonably similar, differences in the flexibility and residual conformations can still exist. Even minor changes may affect the binding activity of a protein: for instance, several authors have shown that post-translational modifications, such as phosphorylation and O-glycosylation, influence the binding profiles of pig OBP isoforms, and phosphorylation can even enhance the binding affinities for some compounds in native OBPs compared to their recombinant counterparts [45,53,54]. It was also hypothesized that the glycosylation pattern of Fel d 1 might affect its structural features, notably by reducing its cavity size, thus possibly altering/modulating its ligand-binding properties [23]. Therefore, we cannot exclude that differences between natural and recombinant forms of Fel d 1 may affect the binding properties of the protein. Confirming our results with the native Fel d 1 would be necessary to definitely assess its putative function as semiochemical carrier.

The proteins that participate in chemical communication have complex roles, such as solubilizing, transporting, serving as reservoirs, assisting in the controlled release of semiochemicals, or even acting themselves as chemical messages (e.g., MUPs) [55,56]. The binding and controlled release of volatile chemical cues via proteins are of particular interest for Felidae, which are mostly solitary carnivores and use scent marks to delimit their territories of variable sizes according to ecological resources [57]. Domestic cats vary greatly in spatial organization, from being solitary in well-dispersed populations at densities of a single individual per square km or lower to living in highly populated groups [58]. Whatever the cats’ social organization is, chemical communication mediated by scent marks is essential to assess social and territorial relationships [59]. The chemical composition of the marks can also provide physiological information in some cases, such as sex or sexual status [60]. Interestingly, other Felidae species also secrete proteins similar to Fel d 1 [61], which might as well have the function of extending the persistence of chemical cues in their environment. Because territory marking involves high energy costs [62], it is important to keep the chemical message as long as possible in general and specifically for Felidae [63].

In mammals, OBPs, sometimes referred to as pheromone-binding proteins (PBPs), are the main proteins that have been reported to mediate chemical communication. These proteins belong to the large family of lipocalins and bind semiochemicals and odorants representing various chemical classes [46,64]. The cat lipocalin Fel d 4 was shown to be involved in chemical communication as a kairomone by eliciting defensive behavior in mice [65]. The structure of secretoglobins (α-helix bundles assembled in a boomerang configuration, creating a central hydrophobic pocket), to which Fel d 1 belongs, is completely different from that of lipocalins (barrel of β-strands with a central apolar cavity) [64,66]. However, the binding data collected with a structural model of Fel d 1 suggest that a function of semiochemical carrier could be considered also for secretoglobins. More experimental evidence is needed, such as studying the expression of Fel d 1 in cat chemosensory organs, confirming its binding activity with the native protein, and perhaps identifying its natural ligands. We hope that our work can stimulate more research in the field of secretoglobins and confirm their putative role in mammalian chemical communication.

Unveiling the ligand-binding properties of Fel d 1 towards semiochemical compounds supports a function of this protein as a semiochemical carrier. As Fel d 1 is one of the most important aeroallergens [4], it is possible that lipid binding might also affect the allergenicity of this protein. Indeed, some authors have shown that another version of recombinant Fel d 1 was able to bind lipopolysaccharides (LPS), enhance lipid cellular signaling through Toll-like receptors, and potentiate the production of the pro-inflammatory cytokine TNF-α (Tumor Necrosis Factor-α), which could eventually influence the allergic sensitization process [67]. In this respect, ligand binding characteristics of Fel d 1 might help to understand the allergenic effects of the protein itself compared to that of its complexes with ligands [68]. Besides, the binding of a ligand to Fel d 1 might affect the allergen recognition by Immunoglobulin E (IgE) if the epitopes are altered through B-cell epitope conformational changes induced by the ligand or if the amino acid residues involved in IgE binding are obscured in the ligand-protein complex. Then, elucidating the ligand binding properties of Fel d 1 might provide valuable insights into this putative phenomenon of ligand-induced epitope masking. Along the same line, several approaches aiming at decreasing or controlling the cat production of immunologically active Fel d 1 have recently been investigated in order to alleviate the symptoms suffered by allergic cat owners [69]. In particular, the use of a diet supplemented with anti-Fel d 1 avian IgY [70] or the immunization of cats with a modified form of recombinant Fel d 1 to stimulate the production of neutralizing antibodies [71] have been proposed. However, as the results of this study suggest that Fel d 1 could play an important role in the cat’s chemical communication, our opinion is that any attempt to alter the production of Fel d 1 should consider possible consequences that might affect the cat’s biology.

## 4. Materials and Methods

### 4.1. System Configuration

All the computational analyses were carried out in a high-performance GPU workstation with Cent OS V.7.6 Linux and the Windows OS. The hardware specifications of the workstation (Model: LVX-1 × RTX-2080Ti) include a powerful Intel Core i9-9920X processor with 1GPU Nvidia RTX-2080Ti, 32GB RAM, running with a superfast boot-home 1 × M2-1TB NVME SSD and 2 × 8TB independent hard disks. The workstation has passed all the validation tests by the Linuxvixion GPU certified system.

### 4.2. Collection and Structure Conversion of Ligands

Molecular structures of the 28 putative ligands (15 fatty acids and their derivatives (FA) and 13 steroids) and N-phenyl-1-naphthylamine (1-NPN) (fluorescent probe) were collected from PubChem (https://pubchem.ncbi.nlm.nih.gov/). All the 2D structures of the ligands were converted into the corresponding three-dimensional (3D) coordinates (sdf to mol2 format) using OpenBabelGUI tools V.2.3.1 (http://openbabel.org). The selected compounds were used to obtain a drug-likeness score from the Lipinski rule of five (RO5) webserver (http://www.scfbio-iitd.res.in/software/drugdesign/lipinski.jsp) [72].

### 4.3. Physio-Chemical Properties Analysis

The physico-chemical properties of all putative ligands and 1-NPN were collected from various chemical databases such as PubChem and ChemSpider. The compound properties were classified as the chemical formula, molecular weight, H-bond donor, acceptor, topological polar surface area, and RO5 (Table 3).

### 4.4. Molecular Docking Analysis

#### 4.4.1. Ligand Optimization

The retrieved molecular structures of the putative ligands and 1-NPN (.mol) were energy minimized using the geometry optimization method (MMFF94 force field) with pH 7.0. The Gasteiger partial charge was added to the ligand atoms and the MMFF94 energies were found to differ between all the compounds. All the nonpolar atoms were merged, and rotatable bonds were defined.

#### 4.4.2. Protein Grid Parameters

The 3D crystal structure of rFel d 1 (PDB ID: 2EJN) was retrieved from the Protein Data Bank (PDB) (https://www.rcsb.org/). The protein dimer and ligand dataset were uploaded to the DockingServer (https://www.dockingserver.com; Virtua Drug, Hungary), a web-based interface module consisting of Gasteiger and PM6 semiempirical quantum-mechanical partial charge calculations to enhance the accuracy of docking output utilizing the AutoDock 4 method [73]. The essential hydrogen atoms, Kollman united atom-type charges, and solvation parameters were added to the 3D structure of rFel d 1. The Gasteiger charge calculation method was selected for the protein clean step. The 3D dimensional grid box was constructed for permitting ligands to interact in the binding sites of Fel d 1. The affinity grid parameters (nx = 23; ny = 23; nz = 23 and cx = −0.48; cy = 0.81; cz = 0.22) and 0.375 Å spacing were generated using the Autogrid program [74]. The total Gasteiger charge of rFel d 1 was −6.959 kcal/mol. After completion of this step, the rFel d 1 structure was prepared for the docking simulation analysis.

#### 4.4.3. Semi-Empirical Calculations

The docking simulation was performed using the Lamarckian genetic algorithm (LGA) and the Solis and Wets local search method to determine the optimum complex [75] in the AutoDock method. The AutoDock parameter set- and distance-dependent dielectric functions were used in the calculation of the van der Waals and the electrostatic terms, respectively. The initial position, orientation, and torsion of the ligand molecules were set randomly, and all rotatable torsions were released during docking. Each docking calculation was derived from 100 runs, which were set to terminate after a maximum of 2,500,000 energy calculations (540,000 for a generation with a population size of 150). A translational step of 0.2 Å, quaternion, and torsion steps of five were employed as parameters for the docking analyses. The AutoDock algorithms calculate the free binding energy to assess the orientation of a ligand binding pose to a protein while forming a stable complex. The protein–ligand complex was analyzed, and the molecular interaction poses of each compound were selected for the ranking of the best-fit ligands according to the docking score with several docking parameters. The estimation of the binding free energy was selected from the best- docked conformation of the protein–ligand complex using docking simulation.

#### 4.4.4. Molecular Visualization

The protein–ligand interactions were visualized using Discovery studio visualizer DSV 4.5 (Accelrys, San Diego, CA, USA), USCF Chimera (https://www.cgl.ucsf.edu/chimera/) and the LigPlot V.4.5.3 program. The evaluation of semi-empirical docking values was computed regarding the score of lowest binding energy, hydrogen bonding (H-bonding), and polar and steric interactions.

### 4.5. Fluorescence Measurement and Binding Assays

N-Phenyl-1-naphthylamine (1-NPN) was used as a non-polar fluorescent probe in competitive binding experiments with the ligands (Sigma, France) to investigate binding efficiency of semiochemical analogs with pure rFel d 1 (INDOOR Biotechnologies, UK) [30]. The fluorescence experiments were performed on an FP-750 spectrofluorometer (JASCO, Japan) instrument at 25 °C in a right-angle configuration with a 1 cm light path fluorimeter quartz cuvette and 5-nm slits for both excitation and emission. The probe 1-NPN was excited at 337 nm and emission spectra were recorded between 380 and 450 nm, at 25 °C. The protein was dissolved in 50 mM Tris-HCl, pH 7.4, and ligands were added as 1 mM methanol solutions.

The rFel d 1 intrinsic fluorescence was expected to be negligible since no tryptophan is present in the sequences of both Fel d 1 chains [48], yet it was verified. The binding of 1-NPN to rFel d 1 was tested at two protein concentrations (1 µM and 2 µM) by titrating the protein solution with aliquots of a 1-mM solution of 1-NPN in methanol to final concentrations of 1–20 µM. The bound ligand was evaluated from the values of fluorescence intensity assuming that the protein was 100% active, with a stoichiometry of 1:1 protein: ligand. Dose–responses curves were performed in triplicate and linearized using Scatchard plots to calculate the 1-NPN dissociation constant (Kd _1-NPN_).

Semiochemicals were first screened for their capabilities to bind rFel d 1 using 1 µM of rFel d 1, 1 μM of 1-NPN, and 2 µM of a competitive ligand. Active compounds were then used to measure their affinity to the protein, using a concentration range of 0–16 μM. The dissociation constants of the competitor ligands (Kd) were calculated from the respective IC50 values (IC50: competitor’s concentration halving the initial fluorescence), using the equation:

Kd = [IC50]/(1 + [1 − NPN]/K_1 − NPN_)

where [1-NPN] is the free concentration of 1-NPN and K_1-NPN_ is the dissociation constant of the complex rFel d 1/1-NPN. IC50 was graphically determined from the dose–response curve of each competitor ligand.

## Figures and Tables

**Table 1:** In silico study of putative molecular residual interactions between recombinant Fel d 1 (rFel d 1) and the compounds and assessment of their capabilities to displace N-phenyl-1-naphthylamine (1-NPN).

n°	Compound Names	Estimated Free Energy of Binding (kcal/mol)	Total Intermolecular Energy (kcal/mol)	Frequency	H-Bond Residue	Hydrophobic Residue (Alkyl/Pi-Alkyl/Pi-Sigma)	In Silico Screening ^a^	1-NPN Displacement Screening
**Fatty Acids and Other Derivatives**								
1	Isobutyric acid	−2.96	−3.26	27%		A88, Y119, L129	No	ND
2	Capric Acid	−5.16	−7.4	23%		L61, F80, V83, F84	No	No
3	Lauric Acid	−5.84	−8.58	60%	Y119	L61, F80, V83, F84	Yes	Yes
4	Myristic Acid	−3.35	−7.02	36%	F84	F13, V133, M134, I137	Yes	Yes
5	Palmitic Acid	−2.33	−5.88	16%	F84	V133, M134	Yes	Yes
6	Oleic Acid	−2.82	−7.05	50%	M134	I64, F80, V83	Yes	Yes
7	Linoleic Acid	−2.95	−6.88	40%	P78	A88, Y119, L129	Yes	Yes
8	Dodecanal	−4.88	−7.42	2%		F84, M134	No	No
9	Dodecanol	−3.93	−7.02	2%		F84, V133, M134	No	No
10	Tetradecanol	−3.97	−7.89	6%		P78, Y81	No	No
11	Ethyl Laurate	−4.7	−8.02	12%	F84	L61, I64, F80, V83, V133, M134, I137	Yes	No
12	Methyl Palmitate	−2.53	−6.67	20%	T76		Yes	No
13	Nonanamide	−4.53	−6.51	4%		L61, I64, V83, F80	No	ND
14	Hexadecanamide	−2.84	−6.3	18%	T135	Y81	Yes	ND
15	Octadecanamide	−2.81	−6.96	6%	G131	Y81, F85	Yes	ND
**Steroids**								
1	Androstenone	−5.84	−5.84	65%	S138	P78, Y81, F85	Yes	Yes
2	Androstenedione	−5.83	−5.83	44%		Y81, F85	Yes	Yes
3	Androstenol	−5.06	−5.36	22%		Y81, F85	No	No
4	Progesterone	−5.74	−6.04	62%	T76	Y81, F85	Yes	Yes
5	Hydroxyprogesterone	−5.14	−5.54	39%	Y81	F85	Yes	Yes
6	Pregnenolone	−5.59	−6.17	58%	T76	Y81, F85	Yes	Yes
7	Estradiol	−4.94	−5.54	26%	T76	Y81, F85	Yes	Yes
8	Testosterone	−5.6	−5.9	35%	T76	Y81, F85	Yes	Yes
9	Dihydrotestosterone	−5.06	−5.35	12%		Y81, F85	No	No
10	Estrone	−3.56	−3.86	10%	D82, G131	F85	Yes	Yes
11	Dehydroepiandrosterone (DHEA)	−4.64	−4.94	30%		Y81, F85	Yes	Yes
12	Corticosterone	−5.35	−6.38	30%	T76, N89	Y81, F85	Yes	No
13	Deoxycorticosterone	−4.99	−5.29	12%		Y81, F85	No	No
**Fluorescent Probe**								
1	1-NPN (Central)	−6.7	−7.41	50%	Y119	L14, L61, M112	Yes	/
2	1-NPN (Surface)	−4.74	−5.45	30%		Y81, F85	Yes	/

ND: Not determined because of the fluorescence increase, probably due to non-specific hydrophobic interactions [42]. ^a^ The in silico screenings were considered to result in positive outcomes (“yes”) if the following were predicted: (1) minimum one H-bond interaction irrespective of the binding frequency or (2) ≥30% of binding frequency without H-bond. This threshold value of binding frequency (≥30%) was selected from the minimum binding frequency of the fluorescent probe (1-NPN) with rFel d 1.

**Table 2:** Affinities of different ligands to rFel d 1, evaluated in competitive binding assays.

Ligand	(IC_50_) (µM)	K_d_ (µM)
Lauric acid	3.3	2.6
Oleic acid	10.0	7.7
Linoleic acid	10.1	7.8
Myristic acid	14.4	11.1
Androstenone	3.1	2.4
Pregnenolone	13.1	10.1
Progesterone	13.6	10.5

**Table 3:** Molecular structural properties of all putative semiochemical compounds and the fluorescent probe N-phenyl-1-naphthylamine (1-NPN).

n°	Compounds	PubChem Compound ID (CID)	Chemical Formula	Molecular Weight (g/mol)	H-Bond Donor	H-Bond Acceptor	Topological Polar Surface Area (Å²)	Lipinski Rule of Five (RO5)
**Fatty Acids and Their Derivatives**								
1	Isobutyric acid	CID_6590	C_4_H_8_O_2_	88.106	1	2	37.3	0
2	Capric acid	CID_2969	C_10_H_20_O_2_	172.268	1	2	37.3	0
3	Lauric acid	CID_3893	C_12_H_24_O_2_	200.322	1	2	37.3	0
4	Myristic acid	CID_11005	C_14_H_28_O_2_	228.376	1	2	37.3	0
5	Palmitic acid	CID_985	C_16_H_32_O_2_	256.43	1	2	37.3	1
6	Oleic acid	CID_445639	C_18_H_34_O_2_	282.468	1	2	37.3	1
7	Linoleic acid	CID_5280450	C_18_H_32_O_2_	280.442	1	2	37.3	1
8	Dodecanal	CID_8194	C_12_H_24_O	184.323	0	1	17.1	0
9	Dodecanol	CID_8193	C_12_H_26_O	186.339	1	1	20.2	0
10	Tetradecanol	CID_8209	C_14_H_30_O	214.393	1	1	20.2	0
11	Ethyl Laurate	CID_7800	C_14_H_28_O_2_	228.376	0	2	26.3	0
12	Methyl palmitate	CID_8181	C_17_H_34_O_2_	270.457	0	2	26.3	1
13	Nonanamide	CID_70709	C_9_H_19_NO	157.257	1	1	43.1	0
14	Hexadecanamide	CID_69421	C_16_H_33_NO	255.446	1	1	43.1	0
15	Octadecanamide	CID_31292	C_18_H_37_NO	283.5	1	1	43.1	1
**Steroids**								
1	Androstenone	CID_6852393	C_19_H_28_O	272.432	0	1	17.1	1
2	Androstenedione	CID_6128	C_19_H_26_O_2_	286.415	0	2	34.1	0
3	Androstenol	CID_101989	C_19_H_30_O	274.448	1	1	20.2	1
4	Progesterone	CID_5994	C_21_H_30_O_2_	314.469	0	2	34.1	0
5	Hydroxyprogesterone	CID_6238	C_21_H_30_O_3_	330.468	1	3	54.4	0
6	Pregnenolone	CID_8955	C_21_H_32_O_2_	316.485	1	2	37.3	0
7	Estradiol	CID_5757	C_18_H_24_O_2_	272.388	2	2	40.5	0
8	Testosterone	CID_6013	C_19_H_28_O_2_	288.431	1	2	37.3	0
9	Dihydrotestosterone	CID_10635	C_19_H_30_O_2_	290.447	1	2	37.3	0
10	Estrone	CID_5870	C_18_H_22_O_2_	270.372	1	2	37.3	0
11	Dehydroepiandrosterone (DHEA)	CID_5881	C_19_H_28_O_2_	288.431	1	2	37.3	0
12	Corticosterone	CID_5753	C_21_H_30_O_4_	346.467	2	4	74.6	0
13	Deoxycorticosterone	CID_6166	C_21_H_30_O_3_	330.468	1	3	54.4	0
**Fluorescent Probe**								
1	N-phenyl-1-naphthylamine (1-NPN)	CID_7013	C_16_H_13_N	219.287	1	1	12	0
